# Task-irrelevant stimuli reliably boost phasic pupil-linked arousal but do not affect decision formation

**DOI:** 10.1038/s41598-024-78791-8

**Published:** 2024-11-17

**Authors:** J. Hebisch, A.-C. Ghassemieh, E. Zhecheva, M. Brouwer, S. van Gaal, L. Schwabe, T. H. Donner, J.W. de Gee

**Affiliations:** 1 Section Computational Cognitive Neuroscience, Department of Neurophysiology and Pathophysiology, University Medical Center Hamburg-Eppendorf, Hamburg, Germany; 2 Cognitive and Systems Neuroscience, Swammerdam Institute for Life Sciences, University of Amsterdam, Amsterdam, The Netherlands; 3 Brain and Cognition, Department of Psychology, University of Amsterdam, Amsterdam, The Netherlands; 4 Amsterdam Brain & Cognition, University of Amsterdam, Amsterdam, The Netherlands; 5 Department of Cognitive Psychology, Institute of Psychology, Universität Hamburg, Hamburg, Germany; 6 Bernstein Center for Computational Neuroscience, Charité Universitätsmedizin, Berlin, Germany

**Keywords:** Cognitive neuroscience, Sensory processing, Human behaviour

## Abstract

The arousal systems of the brainstem, specifically the locus coeruleus-noradrenaline system, respond “phasically” during decisions. These central arousal transients are accompanied by dilations of the pupil. Mechanistic attempts to understand the impact of phasic arousal on cognition would benefit from temporally precise experimental manipulations. Here, we evaluated a non-invasive candidate approach to manipulate arousal in humans: presenting task-irrelevant auditory stimuli at different latencies during the execution of a challenging task. Task-irrelevant auditory stimuli drive responses of brainstem nuclei involved in the control of pupil size, but it is unknown whether such sound-evoked responses mimic the central arousal transients evoked during cognitive computations. A large body of evidence has implicated central arousal transients in reducing bias during challenging perceptual decisions. We thus used challenging visual decisions as a testbed, combining them with task-irrelevant sounds of varying onset latency or duration. Across three experiments, the sounds consistently elicited well-controlled pupil responses that superimposed onto task-evoked responses. While we replicated a negative correlation between task-evoked pupil responses and bias, the task-irrelevant sounds had no behavioral effect. This dissociation suggests that cognitive task engagement and task-irrelevant sounds may recruit distinct neural systems contributing to the control of pupil size.

**Supplementary Information:**

The online version contains supplementary material available at 10.1038/s41598-024-78791-8.

## Introduction

Brainstem arousal systems, including the locus coeruleus noradrenaline system, are transiently (“phasically”) active during the performance of challenging cognitive tasks^1–5^. Pupil dilation at constant luminance is a readily assessable readout of these phasic arousal responses: it is driven by the noradrenergic locus coeruleus, along with other subcortical nuclei involved in arousal, orienting, and alerting, such as the superior and inferior colliculi^4–11^. The pupil also dilates time-locked to challenging perceptual tasks^12–14^, with a time course that reflects the time course of decision formation^5,15^.

Establishing a non-invasive manipulation of phasic, noradrenergic arousal in human cognitive behavior and aberrations thereof is important for both basic and applied science. First, it can be used to pinpoint the causal role of arousal for example in learning or decision-making^1,2,16^. Second, it might lead to clinical approaches to reduce arousal-related cognitive impairments in the context of stress or aging^1,17^. A common assumption underlying causal approaches in neuroscience^18,19^ is that the experimental manipulation induces neural events that precisely mimic those that the brain generates naturally, under physiological conditions. This assumption is typically implicit and rarely tested, especially in human participants for which causal approaches to manipulate brain circuits in a specific fashion are limited to begin with. Thus, experimental manipulations, including those of pupil-linked arousal transients, need to be carefully evaluated.

Task-irrelevant sounds hold promise as a tool for manipulating phasic, noradrenergic arousal during cognitive tasks. Indeed, task-irrelevant sounds accelerate human responses to task-relevant (visual) stimuli^20–25^. Task-irrelevant sounds also cause pupil dilation^25–27^ and drive locus coeruleus activity^6,28–30^. However, other brainstem nuclei also respond to auditory sounds – most strongly the inferior colliculus that is part of the auditory pathway, and whose activity is also read out by pupil size^6^. It is currently unknown if task-irrelevant sounds can be used to reliably manipulate the same central arousal transients that naturally occur during cognitive behavior – specifically, noradrenaline transients.

Here, we evaluated if task-irrelevant sounds can be used to induce phasic pupil-linked arousal responses that mimic those that occur naturally during challenging perceptual decisions. We focused on challenging perceptual decisions because a substantial body of evidence establishes that phasic arousal has a well-understood behavioral consequence in such decisions, namely a reduction of choice bias: (i) intrinsic trial-to-trial variations in the amplitude of task-evoked brainstem and pupil responses (i.e., those that are unrelated to external stimuli or actions) predict a bias reduction across different species and choice tasks^5,31–34^; (ii) systematic variations of the pupil response (due to computational variables such as uncertainty and surprise) affect perceptual decisions in a similar fashion^32,35–37^; (iii) a causal link between locus coeruleus activity and choice bias has been established in mice^3^; and (iv) these empirical findings stand on solid grounds of computational theory^16,38–40^.

Based on previous work^5,31^, we expected that task-evoked pupil responses fluctuate from trial to trial, and that stronger responses of this type predict smaller choice bias. We also predicted that sound-evoked pupil responses would superimpose onto the task-evoked pupil responses. Critically, we reasoned that if task-irrelevant sounds reproduce the (noradrenergic) arousal transients naturally induced by challenging decisions, then the task-irrelevant sounds should also cause a reduction of choice bias. We tested these hypotheses in three experiments, using two different tasks and varying sound parameters.

## Results

**Fig. 1:**
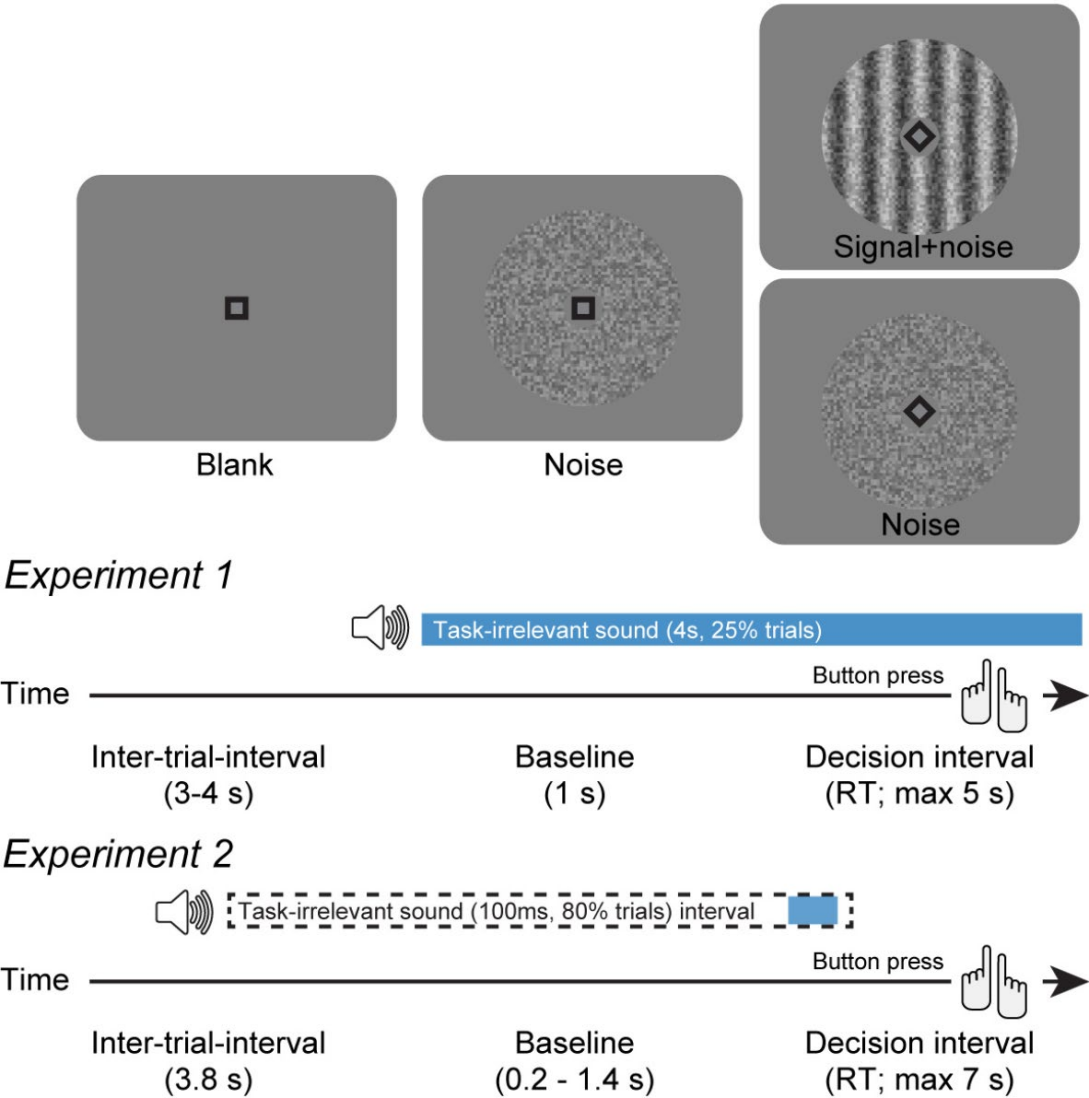
Behavioral tasks. Schematic sequence of events during the simple forced choice task of Experiments 1 and 2. Participants reported the presence or absence of a faint grating signal superimposed onto dynamic noise. Signal contrast is high for illustration only. In Experiment 1, the task-irrelevant, auditory white-noise (70 dB) stimulus started simultaneously to baseline onset (25% of trials). In Experiment 2, brief white noise stimuli (100 ms, 70dB) were played randomly between 3 s before and 0.5 s after decision interval onset (80% of trials).

We first conducted two parallel experiments in which participants made challenging perceptual decisions based on visual information while we varied the frequency, timing, and duration of a task-irrelevant auditory white noise stimulus, henceforth referred to as “task-irrelevant sound” (Fig. 1; see Fig. S1 for task behavior). Previous studies on the behavioral effects of task-irrelevant sounds used different kinds of stimuli with converging results^21–25^. We here chose white noise sounds because they have been shown to affect behavior (accuracy) when presented continuously^41–43^. In Experiment 1, participants reported the presence or absence of a visual Gabor grating of near-threshold contrast superimposed onto flickering visual noise^5^. The task-irrelevant sound started together with a baseline interval of 1 s duration (visual noise only) preceding a so-called decision interval, which either contained the Gabor stimulus or not, and the beginning of which was signaled to the participant. The white noise lasted 4 s and occurred only on 25% of trials. Experiment 2 used the same detection task but was tailored to delineate the time course of the effects of the task-irrelevant sound on behavior at high temporal resolution. Here, task-irrelevant sounds were of short duration (100 ms), occurred on 80% of trials, and started at random times between − 3 s and 0.5 s with respect to decision interval onset (uniformly distributed).

### Trial-to-trial variability of task-evoked pupil responses

**Fig. 2:**
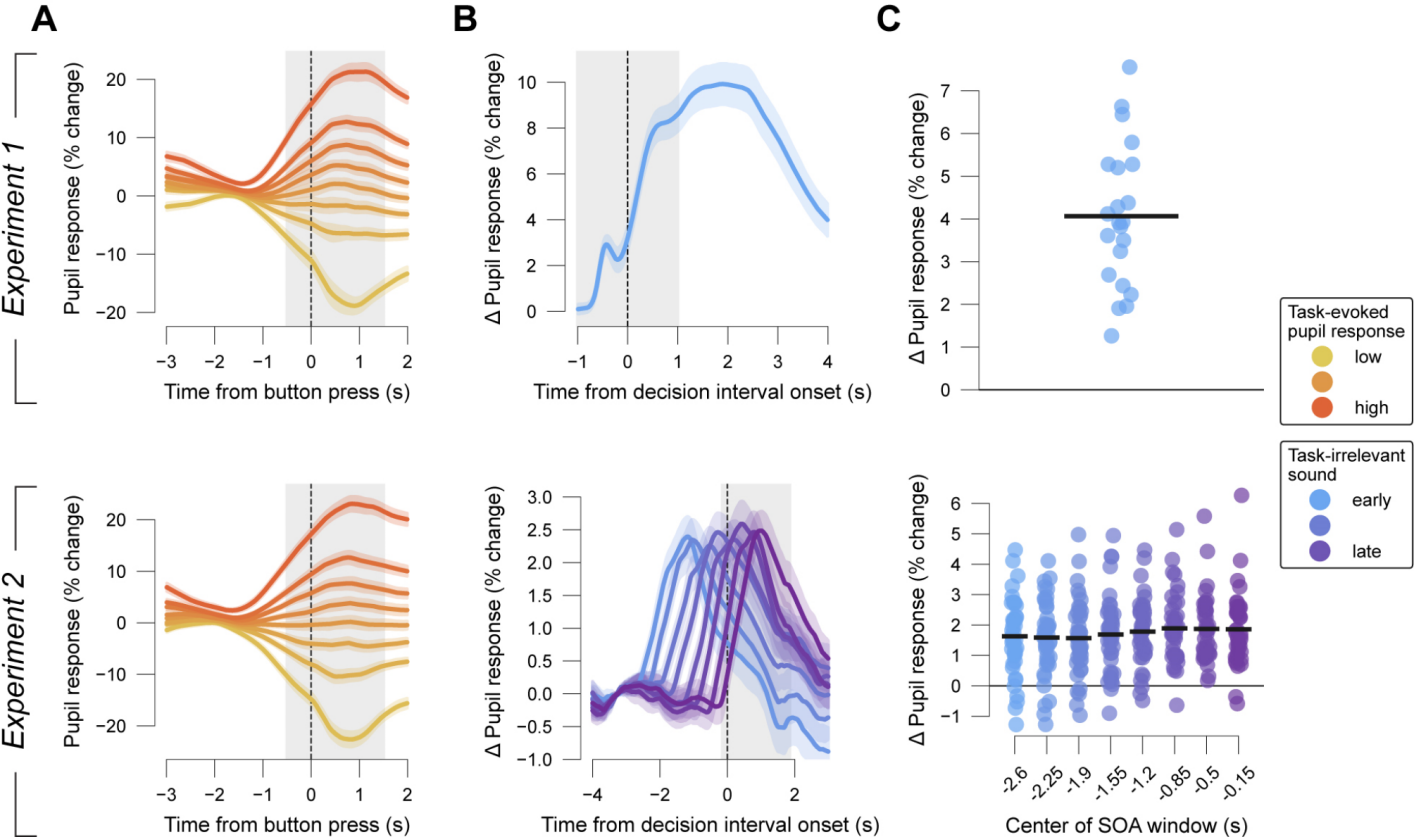
Time courses of pupil responses. (**A**) Pupil response time course on trials with no task-irrelevant sound, binned post-hoc by size of task-evoked pupil response, time-locked to button-press (choice). Shading, S.E.M. across participants. Grey shading, interval used for quantifying task-evoked pupil responses (Methods). (**B**) Differential pupil size time courses between task-irrelevant sound trials and trials without task-irrelevant sound. Baselined with 0.5 s intervals from before the first possible task-irrelevant sound. For Experiment 2, data were binned with a window sliding along possible task-irrelevant sound onset asynchronies (SOAs; window size, 800ms; step size 350 ms). Shading, S.E.M. across participants. Grey shading, interval used for quantifying task-irrelevant sound-evoked pupil responses (bottom, exemplary for last SOA window centered at -0.15 s; Methods). (**C**) Participant-wise task-irrelevant sound-evoked pupil response magnitude. Black bars, mean across participants.

In our experiments, pupil responses during decisions were comprised of a mix of contrast-related constriction at baseline (visual noise) onset^44^ and a dilatory component related to task engagement that built up during decision formation^5,13,15^. Importantly, we observed substantial trial-to-trial variability in the magnitude of the task-evoked pupil responses (Fig. 2A, Methods): bins containing the highest 12.5% of task-evoked pupil responses on trials with no task-irrelevant sound showed a clear dilation (average bin pupil response ± S.E.M.: Experiment 1, 18.625 ± 0.154% signal change; Experiment 2, 19.760 ± 0.292% signal change) whereas those containing the lowest responses showed pupil constrictions (average bin pupil response ± S.E.M.: Experiment 1, -14.342 ± 0.151% signal change; Experiment 2,, -18.548 ± 0.274% signal change). The effect of the near-threshold target contrast (present on a fraction of trials, Fig. 1, top) superimposed on dynamic noise (present in all trials) on pupil responses is negligible^15^. Therefore, we conclude that the trial-to-trial variability of task-evoked pupil responses (time-locked to button press) stems from endogenous sources.

### Task-irrelevant sounds evoke dissociable and reliable pupil responses

We next tested if sound-evoked pupil responses occur in the presence of, and are separable from, task-evoked pupil responses. Thus, we quantified the pupil responses specifically evoked by the task-irrelevant auditory white noise stimuli. To this end, we subtracted the average pupil size time course on trials without a task-irrelevant sound from each trial’s time course with a task-irrelevant sound (Methods). The mean resulting time courses showed robust increases in pupil size with respect to pre-trial baseline, and a return to that same baseline roughly 3–4 s after sound offset (Fig. 2B).

The pupil-response evoked by the long sound (4 s) in Experiment 1 was bi-phasic (Fig. 2B, top). The time-to-peak and height of the first response component was similar to what we observed in Experiment 2 for short auditory stimuli (100 ms): time-to-peak, ~ 700ms; height, ~ 3% signal change. The second response component (in Experiment 1) lasted throughout the 4s long auditory stimulus. The first transient may reflect the presence (possibly combined with unexpectedness) while the second sustained response component may reflect the characteristics (duration and white noise) of the task-irrelevant sound.

We quantified the size of the task-irrelevant sound-evoked pupil response as the mean of this differential time course within a time window of 2 s following task-irrelevant sound onset. We observed a positive effect of task-irrelevant sound on pupil size in virtually every participant (Fig. 2C). Repeated measures ANOVA (i.e., a t-test in case of Experiment 1) revealed a main effect for condition (no-task-irrelevant sound and task-irrelevant sound conditions) on pupil response in both experiments (Experiment 1: t_(21)_ = 11.487, *p* < 0.001; Experiment 2: F_(8, 320)_ = 18.578, *p* < 0.001). Bayesian t-tests additionally indicated that task-irrelevant sounds very likely caused an increase in pupil-linked arousal (Methods) in all timing windows or conditions of all experiments (so across different frequencies of task-irrelevant sound occurrence) given the respective data (BF_10_ range from 2.25e^7^ to 1.23e^11^). The magnitude of task-irrelevant sound-evoked pupil responses was relatively stable over the time course of the experimental blocks, showing no sign of habituation (Fig. S2A) and seemed to largely depend on the duration of the task-irrelevant sound (Fig. 2B, C, compare top to bottom).

In sum, auditory white noise stimuli evoked reliable pupil responses, which were superimposed onto the task-evoked responses and precisely timed.

### Amplitude of task-evoked pupil responses predicts reduction of choice bias

**Fig. 3:**
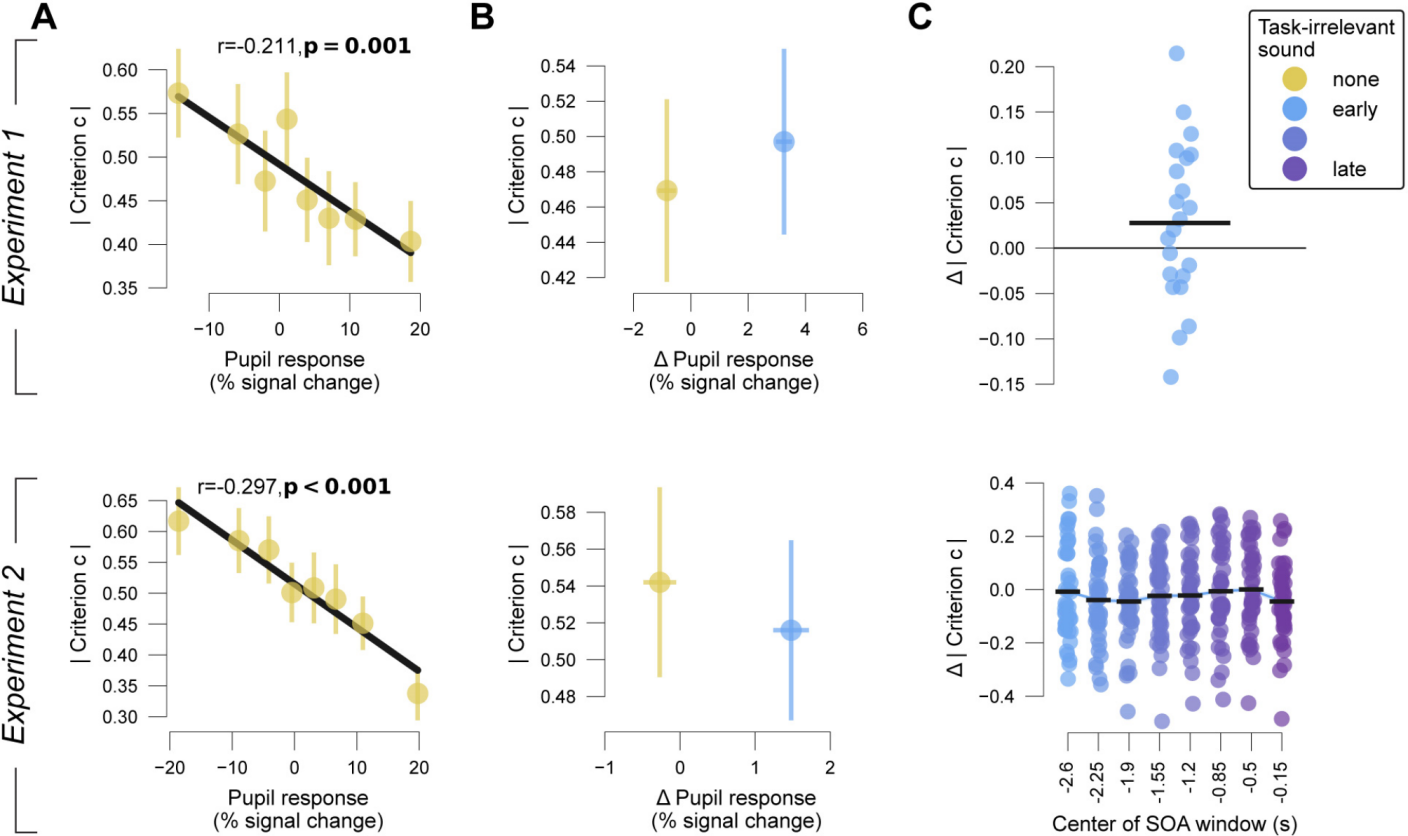
Pupil responses and choice bias. (**A**) Absolute choice bias (criterion c) plotted against task-evoked pupil response for experiments 1 and 2, for trials without a task-irrelevant sound. Error bars, S.E.M. across participants. (**B**) Mean absolute choice bias (criterion c) on trials with and without task-irrelevant sound plotted against mean task-irrelevant sound-evoked pupil responses. Error bars, S.E.M. across participants. (**C**) Participant-wise difference in absolute bias on task-irrelevant sound versus no task-irrelevant sound trials. Black bars, mean across participants.

We then sought to replicate the observation from previous work using the same contrast detection task, which showed a negative correlation between task-evoked pupil responses and bias^5,31^. Participants exhibited a substantial and consistent conservative bias (group average signal detection theoretic criteria ± S.E.M.: 0.381 ± 0.072 and 0.491 ± 0.056 for Experiments 1 and 2, respectively; Fig. S1B). Indeed, the magnitude of task-evoked pupil responses was negatively correlated with (absolute) bias (group average correlation coefficients r: Experiment 1, r=-0.211, t_(21)_=-3.953, p = 0.001; Experiment 2, r=-0.297, t_(40)_=-4.645, p < 0.001; Fig. 3A). We did not observe a consistent relationship between pupil responses and sensitivity (Fig. S3A) or reaction time (Fig. S3C), also in line with earlier work^5,31^. Experiment 1 included two types of experimental blocks that differed in terms of target signal frequency (presence of gratings on 40% vs. 60% of trials). Accordingly^31,45^, participants had a less conservative bias on the “frequent-signal” blocks than on the “rare-signal” blocks (Fig. S4A). However, there was no interaction between block type and the task-evoked pupil response effect on absolute bias (Fig. S4B,C).

### Task-irrelevant sound has no consistent effect on elementary decisions

Having established precise experimental control of pupil responses via the task-irrelevant sounds and having found that task-evoked pupil responses predict a reduction of choice bias, we finally tested if task-irrelevant sounds cause a reduction of choice bias. We found no evidence for that hypothesis (Fig. 3B, C, Experiment 1: t_(21)_ = 1.489, *p* = 0.151; Experiment 2: F_(8, 320)_ = 1.239, *p* = 0.276). The differences in absolute criterion between trials with and without task-irrelevant sound supported the null-hypothesis (BF_10_ ranging from 0.169 to 0.873). Intrinsic variability in task-irrelevant sound-evoked pupil-response also did not correlate with absolute choice bias (Fig. S6).

Task-irrelevant sounds may distract participants from the task at hand, which should prolong decision times and/or reduce evidence sensitivity. In contrast to this prediction, we found no clear and consistent effect of the task-irrelevant sound on sensitivity or reaction time across the two experiments (Fig. S5) with one exception: In Experiment 1 (but not Experiment 2), the task-irrelevant sound reduced both reaction time (∆RT=-0.086 ± 0.077 s, t_(21)_=-5.108, *p* < 0.001, BF_10_ = 546.274) and sensitivity (∆d’=-0.126 ± 0.194 s.d., t_(21)_=-2.984, *p* = 0.007, BF_10_ = 6.652), which is inconsistent with distraction (see above), but indicates a change in the speed-accuracy trade-off towards speed.

Taken together, task-irrelevant sounds had no consistent effect on choice behavior. Specifically, we neither found support for bias-reducing nor distracting effects of task-irrelevant sounds.

### Task-irrelevant sound does not alter weighing of evidence on choice

Our two previous experiments involved simple perceptual decisions about the presence of a visual target signal superimposed on noise. Studies of more complex decisions requiring belief updating in the face of sequentially presented, discrete evidence samples reported that pupil responses predict the weight participants assign to new evidence while updating their beliefs^32,35,46^. To complete the picture of the effects of boosting phasic pupil-linked arousal with results in this more cognitively challenging perceptual decision-making domain, we wanted to know if boosting arousal by means of task-irrelevant sounds results in a similar upweighting of concomitant sensory evidence, or if, as for the null effect on overall choice bias, there is no such effect. To test this, we used a perceptual decision-making task with discrete evidence samples^47^, so that we could readily compute so-called “psychophysical kernels” that quantify the time course of the weighting of sequentially presented sensory evidence on the final behavioral choice^48,49^. Additionally, the placement of category boundaries implied a non-monotonic mapping from sensory (orientation) information to decision-relevant evidence^47^ and rendered the task more challenging than standard perceptual choice tasks.

**Fig. 4:**
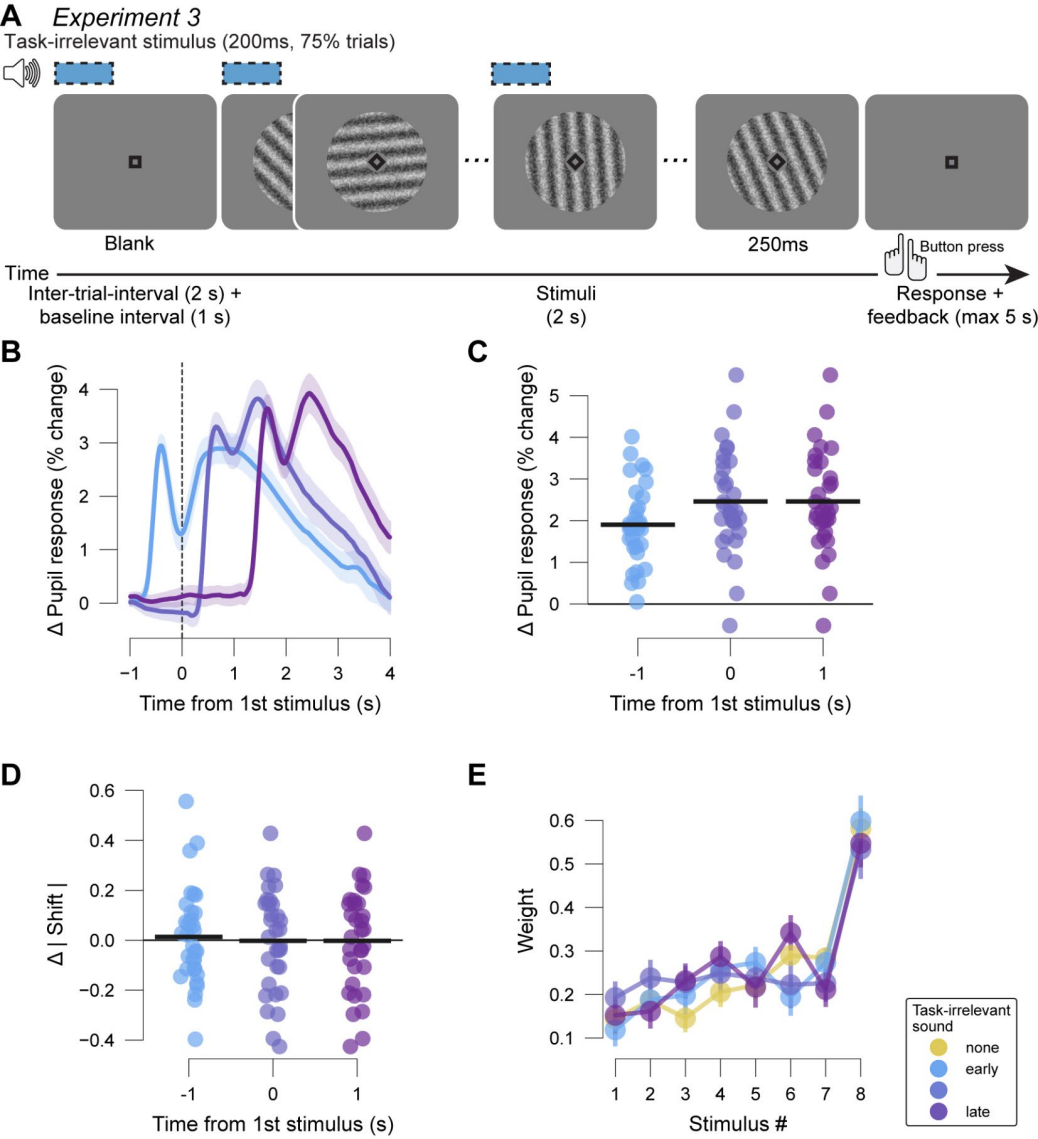
Task design, pupil responses and choice biases in Experiment 3. (**A**) Schematic sequence of events during the category level averaging task of Experiment 3. Participants reported the category (cardinal vs. diagonal) of the average orientation of gratings shown during each trial. White noise stimuli (75 dB) were played at the start of the inter-trial-interval, the first grating or the fifth grating (75% of trials). (**B**) Differential pupil size time courses between task-irrelevant sound trials and trials without task-irrelevant sound by task-irrelevant sound timing. Shading, S.E.M. across participants. Baselined with 0.5 s intervals from before the first possible task-irrelevant sound. (**C**) Participant-wise task-irrelevant sound-evoked pupil response magnitude. Black bars, mean across participants. (**D**) Participant-wise difference in absolute bias (shift of psychometric function) on task-irrelevant sound versus no task-irrelevant sound trials. Black bars, mean across participants. (**E**) Regression weights of visual stimulus position as predictors of choice (psychometric kernels). Error bars, S.E.M. across participants.

In this Experiment 3 (Fig. 4A), participants reported whether the average orientation of a sequence of eight Gabor gratings of varying orientation was closer to the cardinal or diagonal axis^47^. The task-irrelevant sound started either at the same time as the baseline interval, together with the first grating, or together with the fifth grating in the sequence of eight, lasted for 200 ms and occurred at a pseudo-random 75% of trials. The design of Experiment 3 complicated the quantification of intrinsic variations of task-evoked pupil response: each trial contained a sequence of evidence samples of varying strengths, which could elicit arousal responses driven by uncertainty or surprise of different magnitudes within and across trials^35,36^. Thus, in Experiment 3 we did not characterize the intrinsic trial-to-trial variability in task-evoked pupil responses, as we did for Experiments 1 and 2 (Fig. 2A), but focused exclusively on the impact of task-irrelevant sound and the resulting pupil response.

We quantified the pupil responses specifically evoked by the task-irrelevant auditory white noise stimuli in the same way as in Experiments 1 and 2. The mean resulting time courses showed robust increases in pupil size with respect to pre-trial baseline, and a return to that same baseline roughly 3–4 s after sound offset (Fig. 4B). A repeated measures ANOVA revealed a main effect for condition (no-task-irrelevant sound and task-irrelevant sound conditions) on pupil response (Experiment 3: F_(3,93)_ = 71.742, *p* < 0.001; Fig. 4C). Bayes factor supported that task-irrelevant sounds very likely caused an increase in pupil-linked arousal in all timing conditions (BF_10_ range from 5.5e^9^ to 2.35e^11^).

Participants exhibited a substantial and consistent bias towards the diagonal choice category (group average shift of psychometric function ± S.E.M.: 0.36 ± 0.059). Task-irrelevant sounds, however, did not change absolute choice bias (Fig. 4D; F_(3, 93)_ = 0.313, *p* = 0.816; BF_10_ for differences ranging from 0.189 to 0.232), nor sensitivity (slope of psychometric function; Fig. S7B; F_(3, 93)_ = 1.059, *p* = 0.37; BF_10_ for differences ranging from 0.267 to 1.173).

We also estimated the weighting of evidence samples on choice using logistic regression (Methods). On trials without a task-irrelevant sound, participants exhibited an overall recency bias: a tendency to rely more on the most recent sample(s) of evidence (Fig. 4E). Critically, the psychophysical kernels on task-irrelevant sound trials were not different from the one computed on trials without a task-irrelevant sound (Fig. 4E; Wilcoxon uncorrected p-values ranging from 0.071 to 0.905). Taken together, task-irrelevant sounds also did not seem to alter the overall bias nor evidence weighing in this more complex task.

### No choice effect of task-irrelevant sound dependent on baseline pupil size

Having established an absence of behavioral effect of task-irrelevant sounds on different perceptual decision-making tasks, we finally tested for interaction of task-irrelevant sound effects on the pupil and choice bias with baseline pupil size. The amplitudes of task-evoked and task-irrelevant sound-evoked pupil responses were roughly stable across trials within blocks (Fig. S2B,** C**). By contrast, we observed large changes of the pre-trial baseline pupil sizes throughout the experiment, with a gradual constriction early in the block down to an asymptotic level (Fig. S2A). This was unlikely due to light adaptation, since participants sat in the same dark room for at least 5 min prior to the start of the experiment blocks, and because in one experiment (Experiment 2) with multiple longer (10 s duration) breaks between blocks of 100 trials, the pupil size dilated again in each break, resetting the process of monotonic constriction during the next block, despite constant illumination levels (Fig. S2A, middle).

**Fig. 5:**
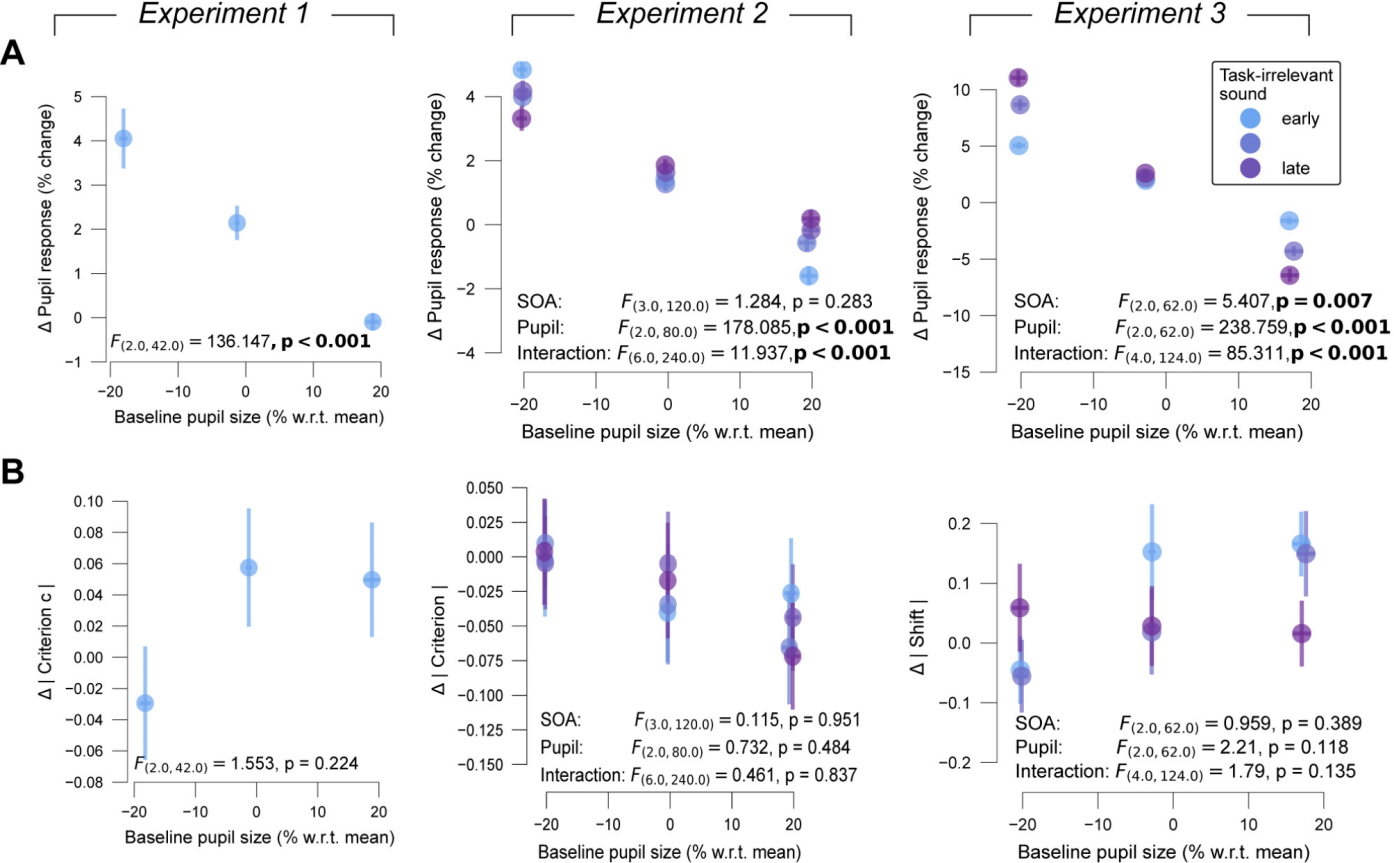
Effects of task-irrelevant sound as function of pre-trial baseline pupil size. (**A**) Task-irrelevant sound-evoked pupil response plotted against pupil baseline size (binned post-hoc by size). Shading, S.E.M. across participants. Statistical metrics, results of repeated measures ANOVA. (**B**) As (**A**) but for difference in absolute bias (criterion c, shift of psychometric function) between trials with and without task-irrelevant sound.

As expected from previous work^15,31^, pupil responses evoked by the task-irrelevant sound were negatively related to pre-trial baseline pupil size (*p* < 0.001 on all experiments; Fig. 5A). Critically, however, differences in choice bias between task-irrelevant sound trials and those without did not depend on the pre-trial baseline size of the pupil (Fig. 5B). As pre-trial pupil sizes may contain spill-over from previous evoked pupil responses from the previous trial, we also assessed the baseline pupil diameter in the break intervals in Experiment 2. We found no effect of pupil size during these intervals on task-irrelevant sound effects on absolute bias (F_(3,120)_ = 0.33, *p* = 0.804) or task-irrelevant sound-evoked pupil response on the following 100 trials (F_(3,120)_ = 0.234, *p* = 0.873). There was also no interaction between effects of task-irrelevant sound timing window and pupil size during the break intervals on task-irrelevant sound effects on absolute bias (timing window: F_(3,120)_ = 0.358, *p* = 0.784; interaction: F_(3,120)_ = 0.48, *p* = 0.888) or task-irrelevant sound-evoked pupil response on the following 100 trials (timing window: F_(3,120)_ = 1.21, *p* = 0.309; interaction: F_(3,120)_ = 1.615, *p* = 0.109).

## Discussion

For establishing the applicability of task-irrelevant sounds as a manipulation of phasic pupil-linked arousal, it is necessary to assess whether this manipulation triggers arousal responses that mimic those that occur naturally. Here, within the same participants, we systematically compared the response profiles and behavioral correlates of phasic pupil-linked arousal responses that were task-related with those induced by task-irrelevant sounds. We expected the latter to drive phasic responses of arousal-controlling brainstem nuclei, including the noradrenergic locus coeruleus^6,28–30^. We conducted three experiments, in which participants performed challenging perceptual decisions while we varied the frequency, timing, and duration of the task-irrelevant sound. We replicated the negative correlation between intrinsic trial-to-trial variations in task-evoked pupil response amplitude and choice bias established in several previous studies^5,31–34^. As expected, the pupil did not only dilate during task engagement but also in response to the white noise sounds. All variants of this task-irrelevant sound drove robust and precisely timed pupil responses that were superimposed onto the task-evoked pupil responses and were separable through a simple linear subtraction. Yet, in neither experiment did these task-irrelevant sounds reduce choice bias, nor did we observe any other consistent behavioral effect.

The task-irrelevant sound-evoked pupil responses were smaller in amplitude than the range of intrinsic trial-by-trial variations of task-evoked pupil responses. Yet, the sound-evoked pupil responses were substantial in magnitude and reliable across subjects. Since the relationship between phasic pupil-linked arousal and choice bias is linear across the arousal’s full dynamic range (Fig. 3A**)**^5,31^, even a modest change in phasic arousal could, potentially, affect bias. Therefore, the smaller amplitude of sound-evoked compared to task-related pupil-linked arousal is unlikely to account for the complete absence of a behavioral correlate.

One previous study reported that task-irrelevant sounds can reduce choice bias^50^. However, in that task the auditory white noise was terminated upon the participant’s choice (button press). Thus, participants could control the offset of the slightly aversive sound stimulus, which may have caused them to favor speed over sensitivity, and which may have reduced choice bias. We took care to avoid this confound, and in three separate experiments did not find a consistent effect of task-irrelevant sounds on choice bias or other decision-making metrics.

Why did task-irrelevant sounds not influence decision-making despite producing reliable pupil responses? The interpretation depends on the assumption about the similarity of the central arousal events driven by the task and those driven by the task-irrelevant sound, which is currently unknown. If the two forms of central arousal events were identical (i.e., identical activation pattern of arousal-controlling brainstem systems), our result would imply that the correlation between trial-to-trial variations in task-evoked pupil responses and choice bias does not reflect a causal effect of arousal on behavior. For example, it is possible that forming a decision against one’s “default” choice option drives arousal. The latter could be conceptualized as a high-level form of surprise, akin to updating one’s internal belief with evidence that contradicts the prior belief, which drives pupil dilations^35,37,46,51,52^. The time course of the correlation between task-evoked pupil responses and bias in reaction time versions of detection tasks shows that this relationship emerges long (~ 800 ms) before a choice is reported^5^. Due to the delay of the pupil response relative to brainstem activity^6^, the underlying central arousal transient must have occurred even earlier. While it is possible that the content of evolving decisions per se changes central arousal state, it seems difficult to explain early pupil-behavior correlations by such (secondary) activation.

An alternative possibility is dissimilarity of the central arousal events recruited by task-irrelevant sounds and by the task. Evidence from fMRI suggests that task-evoked arousal and specifically pupil-linked bias reduction is mainly due to responses of neuromodulatory brainstem nuclei, rather than the superior and inferior colliculi^5^. Task-irrelevant sounds, on the other hand, seem to elicit smaller responses in the locus coeruleus compared to the inferior colliculus^6^, which is a key structure in the central auditory pathway. Task-irrelevant stimuli might also recruit different sub-populations of neurons within the locus coeruleus than cognitive engagement. The locus coeruleus is more heterogeneously organized than long assumed^53,54^. While the locus coeruleus is activated, to some extent, by both task engagement and sounds^1,5,6,28–30,55^, the relative amplitudes and neuronal sub-populations of these two types of locus coeruleus responses are unknown. Finally, multiple brainstem structures involved in the control of pupil size are strongly connected, providing a scaffold for complex interactions. For example, the locus coeruleus and the ventral tegmental area exhibit a close interplay^56,57^ and are co-activated with pupil responses during decisions^5^.

In sum, pupil responses recruited by task engagement (varying in amplitude from trial to trial) and those driven by task-irrelevant stimuli may be determined by the relative contributions of several structures within the brain’s machinery controlling arousal state, with potentially different behavioral consequences. This idea relates to the conceptual distinction between arousing, alerting, and orienting^11,53,58^. For example, distinct functional processes have been postulated for executive, orienting, and alerting of attention^59,60^, whereby executive attention resembles the task recruitment we measured here, while alerting attention is a response to temporal cues, resembling our task-irrelevant sound.

Part of the task-evoked pupil responses quantified here (time-locked to behavioral report) may be driven by the motor execution. Pupil dilation also occurs time-locked to button presses in the absence of a task^61,62^ and is diminished for covert decisions that are not reported through a motor response^61,63,64^. Importantly, the time course of the task-evoked pupil response evoked by our detection task, as well as the time course of the correlation between that pupil response and behavioral bias both speak for an important intra-decisional process^5^.

While the pupil is likely to dilate in response to any sound, the level of unexpectedness of a task-irrelevant sound may be an important factor governing its behavioral effect. Indeed, in our experiments, the frequency of occurrence of the task-irrelevant sounds varied substantially (between 25% and 80%), and we found robust sound-evoked pupil responses throughout. Likewise, sounds that are completely predictable in terms of timing and content still drive pupil dilation^65^. This is expected simply because sounds elicit responses across the auditory pathways up to the inferior colliculus, which, in turn, can dilate the pupil^6^. By contrast, surprising events drive pupil responses in a more cognitive manner^35,37,46,64,66–68^, including even responses to the absence of a sensory change, when change is expected^46^. This high-level component of the phasic arousal response may be critical for recruiting the locus coeruleus. It will be instructive to unravel the importance of these, and other characteristics (e.g., the nature of the sound) of task-irrelevant stimuli for producing central arousal transients that best mimic those driven by cognitive computation.

Previous work has reported that task-irrelevant stimuli produce a reduction in reaction time during cognitive tasks^20,22–25^. Because of this convergent finding, the task-irrelevant stimuli in this literature were typically called “accessory stimuli”. We did not observe a consistent reduction of RT across all our current experiments. Only the least frequent but relatively long task-irrelevant sound in Experiment 1 (25% of trials; 4 s) caused speedier and less sensitive decision-making. The absence of an effect in Experiment 2 and 3 might be explained by the missing temporal binding, typically seen in the accessory stimulus literature. It may also be due to the relatively high frequency of trials on which an accessary stimulus occurred (80% and 75%, respectively), yielding the task-irrelevant sound to be a less surprising outcome. Yet another possibility is that our participants made challenging decisions that required protracted evidence accumulation, while the accessory stimulus literature mostly describes easier tasks that produced significantly shorter RTs^20,22–25^.

Across all experiments reported here, the pupil dilated reliably in response to the white noise bursts, regardless of timing, frequency, or time on task at which they were delivered. The mean value of the dilations seemed to scale with stimulus duration (although these durations were so far only varied across experiments that also differed in other variables) and the dilation latency precisely reflected the stimulus onset latency. This comprehensive assessment of pupil responses to task-irrelevant sounds complements and extends previous work in important ways^25–27^. For example, Petersen et al.^27^ demonstrated the dependence of the pupil response amplitude on sound intensity, while we here demonstrated the dependence on sound duration and/or frequency, providing more room for future experimental manipulation (the permissible range of sound pressure levels in human experiments is quite limited). Most importantly, our work demonstrates that the pupil responses to task-irrelevant sounds can be readily isolated from the task-evoked responses using a simple linear subtraction approach as commonly used in fMRI studies. Like fMRI, this approach ignores possible non-linear interactions between the two types of superimposed arousal responses, which should be tested in future experiments. Yet, the clear stimulus dependence of the resulting estimates of the sound-evoked responses provides strong evidence for an approximately linear superposition.

We found that phasic pupil responses to our manipulation were reduced with increasing baseline pupil size. However, taking baseline pupil size into account did not change any results of decision-making behavior. Nassar et al.^52^ found effects of task-irrelevant sound-switches on learning rate that depended on the baseline pupil size. It is unclear whether our results can be compared to these findings as the behavior in question and the manipulations were different. Additionally, baseline pupil measures used for quantifying tonic arousal are often taken from short inter-trial-intervals and may thus be confounded by spillover of phasic pupil responses from previous trials (but see^69^). We therefore chose to additionally use the purer average pupil size during small intervals of rest (10s) that appeared every 100 trials in Experiment 2. Pupil size during these intervals predicted neither behavioral nor pupil responses to the task-irrelevant sound.

To conclude, establishing approaches for non-invasively manipulating phasic arousal would in principle allow for causal tests of the role of central arousal transients in cognition and could be translated to practical applications in health and disease. For example, aberrancies in the central arousal system and in pupil responses are found in important neuropsychiatric disorders such as Parkinson’s Disease and Alzheimer’s Dementia^70–72^. Here we critically evaluate one such candidate tool, task-irrelevant sounds presented during a challenging task. While we show that these sounds evoked one component of phasic arousal associated with pupil responses, the complexity of the central arousal system calls for careful consideration. This is in line with the emerging view that the brainstem system controlling central arousal state as well as pupil size is likely heterogenous, made up of different sub-systems with distinct functional roles. Future neuroimaging or direct recording studies should further illuminate and dissociate sources of these task-irrelevant stimulus-evoked and task-evoked arousal responses.

## Methods

### Participants

27 healthy participants (13 female; age range, 18–28 y) took part in Experiment (1) 41 healthy participants (29 female; age range, 18–35 y) took part in Experiment (2) 32 healthy participants (19 female; age range, 18–33 y) took part in Experiment (3) All participants had normal or corrected-to-normal vision. We used the effect sizes reported in Bruel et al.^50^ for a power analysis. Based on the magnitude of sound-evoked pupil responses in Fig. 2E of that preprint and a power of 0.8, we required sample size for detecting such an effect of *N* = 5. We used sample sizes much larger than that to ensure that neither of our experiments was underpowered to reproduce such effects (e.g. because pupil responses to briefer sounds would likely be smaller), and also because the effect size of possible relationships to choice bias are unknown. As we conducted repeated measures analyses, we also ensured power by using a sufficiently large number of trials per participant (> 1000), similar to that employed by Bruel et al.^50^.

All participants gave written informed consent and were remunerated by the hour (Experiment 2) or received credit points (Experiments 1 and 3). All experiments were conducted in accordance with the Declaration of Helsinki. Experiments 1 and 3 were approved by the ethics committee of the Department of Psychology at the University of Amsterdam, and Experiment 2 by the ethics committee of the Faculty of Psychology and Human Movement Sciences at the University of Hamburg. Five participants were excluded from Experiment 1 due to low number of trials (< 1000) after removing trials with more than 20% missing pupil data (see analysis of pupil responses) or reaction times below 200 ms or longer than 4.5 s leading to a total sample of 95 participants.

### Behavioral tasks

All participants were asked to sit in front of the screen at 50 cm distance resting their head on a chin rest. They indicated their choice with a button press: ‘z’ or ‘m’ on a keyboard, counterbalanced across participants. The screens and audio equipment used depended on the local facilities at which the experiments took place.

### Contrast detection task (experiments 1 & 2)

Each trial of the contrast detection task^15^ consisted of an inter-trial interval (ITI), a baseline interval, and a decision interval. The ITI showed a blank grey screen and a fixation square. It lasted 3–4 s in Experiment 1 (uniformly distributed) and 3.8 s in Experiment 2. The baseline interval (Experiment 1, 1 s; Experiment 2, 0.2–1.4 s, exponentially distributed) additionally showed flickering visual noise (refresh rate: 100 Hz) in a gaussian annulus around the fixation square with 20% contrast. The decision interval was cued by a 45° rotation of the fixation square, and, on a fraction of trials showed a signal (Gabor grating; 2 cycles per degree; vertical orientation) superimposed onto the visual noise. In Experiment 1, in ‘rare blocks’, the signal appeared in 40% of trials, and in ‘frequent blocks’ on 60% of trials. In Experiment 2, the signal appeared in 50% of trials. Participants reported the presence or absence of the signal with a button press which ended the trial. The maximum possible duration of the decision interval was 5 s in Experiment 1 and 7 s in Experiment 2. Luminance levels were kept stable throughout the whole task. In Experiment 1, visual stimuli were displayed on a gamma-corrected monitor (spatial resolution of 2560 by 1440 pixels) which had a vertical refresh rate of 100 Hz and stood in a dimly lit room (10 Lux), and in Experiment 2 on a VIEWPixx monitor (1920 by 1080 pixels) with a refresh rate of 100 Hz in a dark room (0 Lux).

A task-irrelevant sound (auditory white noise) occurred at a pseudorandom fraction of trials: Experiment 1, 25%; Experiment 2, 80%. In Experiment 1, the task-irrelevant sound (70 dB) started together with the baseline interval and lasted 4 s. In Experiment 2, the task-irrelevant sound (70 dB) was played randomly between 3 s pre and 0.5 s post decision interval onset (uniformly distributed) and lasted for 100 ms. In Experiment 1, task-irrelevant sounds were played from Logitech speakers, and in Experiment 2 through AKG K72 headphones.

Experiment 1 was conducted at the University of Amsterdam over three separate sessions. In the first session (~ 1 h), participants were acquainted with the contrast detection task. We titrated individual task difficulty to about 75% accuracy by adjusting contrast levels of the target stimulus. In each of the sessions two and three (~ 2.5 h each), participants underwent two blocks of the experiment (40 min, 360 trials per block) during which their EEG was recorded, and their eyes were tracked.

Experiment 2 was conducted at the University Medical Center Hamburg-Eppendorf. Participants came into the lab on two days within a week for two hours each day. The first day started with a contrast orientation discrimination task. In this task, the stimulus contrast changed following a staircase procedure aimed at 75% accurate performance. The last contrast level was used as first contrast level of the signal in the contrast detection task. Participants were then asked to perform 5-minute practice blocks of the contrast detection to get acquainted with it and to allow for further manual adjustment of contrast. After four to seven practice blocks, participants took a break and then continued with the first experiment block. On the second day, participants first performed another practice block and then performed two experiment blocks separated by a 5-minute break. Experiment blocks consisted of 400 trials, took approximately 45 min, and included eye tracking.

### Orientation averaging task (experiment 3)

Each trial of the category level orientation averaging task^47^ consisted of an ITI, a baseline interval, a grating sequence interval, and a decision interval. The ITI showed a blank grey screen and a fixation square. It lasted 2 s. The baseline interval was identical and lasted 1 s. During the grating sequence interval, 8 Gabor gratings of individual orientation were shown consecutively for 250 ms each. The decision interval started with the end of the last grating and showed a grey screen with the fixation square. Participants reported whether the average orientation of the gratings belonged to the cardinal or diagonal category (true distribution 50%/50%) with a button press. A pair of tones played 250 ms after their button press served as feedback which ended the trial (correct: 440 followed by 880 Hz, incorrect: 880 followed by 440 Hz). Difficulty level was kept around 75% accuracy with the help of an online staircase procedure adjusting the distance between the angle of grating orientation and the respective perfect cardinal or diagonal angle. Luminance levels were kept stable throughout the whole task. Visual stimuli were displayed on a gamma-corrected monitor (spatial resolution of 2560 by 1440 pixels) with a vertical refresh rate of 100 Hz. The experiment took place in a dimly lit room (10 lx).

The task-irrelevant sound (auditory white noise) occurred at a pseudorandom 75% of trials in this experiment. It was 75 dB loud, 200 ms long, and started either at the onset of the baseline interval, simultaneously to the first Gabor grating or at the same time as the fifth Gabor grating (25% of trials each). Task-irrelevant sounds were played from Logitech speakers.

Experiment 3 was conducted at the University of Amsterdam in two sessions (~ 2 h each). In session one, participants completed two practice blocks (~ 10 min each) before running three experiment blocks (~ 25 min each; 200 trials). In session two, four more experiment blocks took place.

### Eye data acquisition

Eye data were obtained with Eyelink 1000 devices (SR Research, Osgoode, Ontario, Canada) at 1000 Hz with an average spatial resolution of 15 to 30 min arc. The eye trackers were calibrated at the start of each experiment block. In Experiments 1 and 3 we recorded the left eye, and in Experiment 2 we recorded the right eye (depending on the local researcher). The recording of one eye is standard practice in this field, because pupillary responses are consensual^73,74^: the two pupils react simultaneously and by the same amount in all but pathological cases.

### Analysis of pupil responses

#### Preprocessing

Pupil data were analyzed with custom-made Python scripts like those used in previous studies^5,15^. Blinks and saccades were detected by the manufacturer’s standard algorithms. Missed blinks were detected with a custom algorithm^75^. Pupil data were low-pass filtered with a third-order Butterworth filter with a cut-off at 10 Hz to correct for random fast fluctuations in pixels recognized as pupil. Missing data (e.g. caused by blinks) were linearly interpolated in a window of 200 ms before up to 200 ms after the missing data. Pupil responses to blinks and saccades were corrected using a double gamma function convolution^65^. Using the median, the units of the pupil size time series were transformed from pixels to percent signal change.

### Quantification of task-evoked pupil responses

To measure trial-wise task-evoked pupil responses, we created epochs centered on choice (button-press) for trials with no task-irrelevant sound. Epochs with more than 20% missing data (due to blinks or general signal loss) were excluded from the analyses. The epoched pupil size data was baselined with the mean of half a second before the first visual target stimulus could appear (decision interval onset). For each trial, we then calculated the mean (baseline subtracted) pupil size over two seconds starting 0.5 s before choice (button-press)^15^. To specifically isolate trial-to-trial variations of underlying response amplitudes, variations due to baseline pupil size and reaction time were removed from the task-evoked pupil responses (via linear regression)^5^. In one analysis, trials were sorted by task-evoked pupil response amplitude and collapsed into eight bins (Fig. 2A).

### Quantification of task-irrelevant sound-evoked pupil responses

For measuring the pupil responses evoked by the task-irrelevant sound in Experiment 1 and 3, we created epochs centered on task-irrelevant sound onset. Epochs with more than 20% missing data (due to blinks or general signal loss) were excluded from the analyses. Epochs were baselined using the mean pupil size from half a second before task-irrelevant sound onset. From each epoch, we then subtracted the block-wise average pupil time series on the trials without a task-irrelevant sound. For experiment 2, we used epochs centered on decision-interval onset. These were baselined using the mean pupil size from half a second before the first possible onset of task-irrelevant sounds. From these epochs, we subtracted the block-wise average pupil time series on the trials without a task-irrelevant sound. Epochs were then aligned to task-irrelevant sound onset and baselined again with the mean pupil size from half a second before task-irrelevant sound onset. For all experiments, we then calculated the mean (baseline subtracted) differential pupil size over two seconds following task-irrelevant sound onset for each trial.

### Quantification of baseline pupil size

Pre-trial baseline pupil size was quantified as the mean pupil size (percent signal change) over 0.5 s before the first possible event in each trial. Experiment 2 included five 10 s-intervals in each block (every 100 trials). From these we extracted longer baseline values by averaging pupil size over 7 s starting 2 s after interval onset.

### Analysis of choice behavior

For all analyses, we excluded the first 10 trials of each block due to habituation effects (see Fig. S2).

In experiments 1 and 2, we computed signal detection theory metrics sensitivity d’ and criterion c^45,76^. The sensitivity describes the participants’ ability to discriminate signal from noise and is calculated as the difference between the z-scored hit rate and false alarm rate. The choice bias describes the participants’ intrinsic tendency to prefer one choice alternative over the other and is calculated as the average of z-scored hit and false alarm rates multiplied by -1.

In Experiment 3, we used the slope and shift from a fitted psychometric function. The psychometric function was obtained from a logistic regression that used the normalized sample-wise (signed) evidence strength (i.e. orientation of each of the trial’s eight Gabor gratings) to predict choice. The average regression coefficient of the Gabor gratings quantified the slope reflecting sensitivity. The regression intercept quantified the shift reflecting choice bias. Similarly, psychophysical kernels were quantified as regression coefficients of sample-wise (signed) evidence strength (i.e. orientation; not normalized).

In Experiment 1 and 2, reaction time was defined as the time from decision interval onset until the button press. For analysis, these values were collapsed into 3 bins. In Experiment 3, reaction time was defined as the time from offset of the final stimulus in the sequence until the button press. Trials with reaction times lower than 0.2 s or higher than 4.5 s were excluded from analyses.

### Statistical comparisons

For each participant in Experiments 1 and 2, we computed the Pearson correlation between each of the behavioral metrics and the bin-wise average task-evoked pupil response. The sample of correlation coefficients was then compared against 0 with a paired-samples t-test. For Experiment 1, all metrics were calculated by block type and then averaged (Figs. 2C and 3C) or tested separately (Fig. S4).

We compared pupil size and behavioral metrics on trials with a task-irrelevant sound versus trials without. In Experiment 2, we binned trials with a sliding window along the possible task-irrelevant sound onset times (length, 800 ms; step size, 350 ms). In Experiment 3, we considered the three possible task-irrelevant sound onset times separately. We assessed the task-irrelevant sound effect with repeated measures ANOVAs with (no-)task-irrelevant sound condition (or window) as within-subject factor. Further, differences between each task-irrelevant sound condition or time window and the no task-irrelevant sound condition was tested against zero using Bayesian t-tests (Figs. 2C, 3C and 4C and D, S5).

For analysis of the psychophysical kernels in Experiment 3, we tested each kernel of each condition with a task-irrelevant sound against the respective kernel of the condition without a task-irrelevant sound using FDR-corrected Wilcoxon tests (Fig. 4E).

We checked for interaction of task-irrelevant sound effects behavior and pupil response with pre-trial baseline pupil size by means of repeated measures ANOVA. We tested the effects of the within-subject factor pre-trial pupil baseline bin on task-irrelevant sound-evoked pupil response or on difference in absolute bias towards the condition without task-irrelevant sounds (Fig. 5). For Experiments 1 and 3, this ANOVA also included the factor task-irrelevant sound condition (i.e., task-irrelevant sound SOA time windows in Experiment 2). Additionally for Experiment 2, we calculated an ANOVA testing the effects of the 10-s break interval pupil size (as well as task-irrelevant sound SOA window) on the effects of the task-irrelevant sound on absolute bias or pupil response in the following 100 trials. For this analysis, task-irrelevant sound SOAs were binned into 4 bins with cut-offs at -2.125 s, -1.25s, and − 0.375 s with regard to decision interval onset.

## Electronic supplementary material

Below is the link to the electronic supplementary material.

Supplementary Material 1

## Data Availability

The datasets generated and analyzed during the current study can be found online at https://www.fdr.uni-hamburg.de/record/15976. Analysis scripts are available under https://github.com/jhebisch/2024_Hebisch_task-irrelevant-sounds-boost-pupil-no-influence-on-behavior.
