# Supplementary material for: Task-irrelevant stimuli reliably boost phasic pupil-linked arousal but do not affect decision formation
Source: Sci Rep. 2024 Nov 17;14:28380. doi: 10.1038/s41598-024-78791-8 (PMC11570621; doi:10.1038/s41598-024-78791-8)
Supplement: Supplementary file 1 — Supplementary Material 1 [file 41598_2024_78791_MOESM1_ESM.pdf]

Task-irrelevant stimuli reliably boost phasic pupil-linked arousal but do not affect decision formation

J. Hebisch[1,*], A.-C. Ghassemieh[1], E. Zhecheva[2], M. Brouwer[2], S. van Gaal[3,4], L. Schwabe[5], T.H. Donner[1,6,#,*] & J.W. de Gee[2,4,#,*]

[1]Section Computational Cognitive Neuroscience, Department of Neurophysiology and Pathophysiology, University Medical Center Hamburg-Eppendorf, Hamburg, DEU; [2]Cognitive and Systems Neuroscience, Swammerdam Institute for Life Sciences, University of Amsterdam, Amsterdam, NLD; [3]Brain and Cognition, Department of Psychology, University of Amsterdam, Amsterdam, NLD; [4]Amsterdam Brain & Cognition, University of Amsterdam, Amsterdam, NLD; [5]Department of Cognitive Psychology, Institute of Psychology, Universität Hamburg, DEU; [6]Bernstein Center for Computational Neuroscience, Charité Universitätsmedizin, Berlin, DEU.

# = Equal contribution

∗ = Address correspondence to: j.hebisch@uke.de , t.donner@uke.de and j.w.degee@uva.nl

SUPPLEMENTARY FIGURES

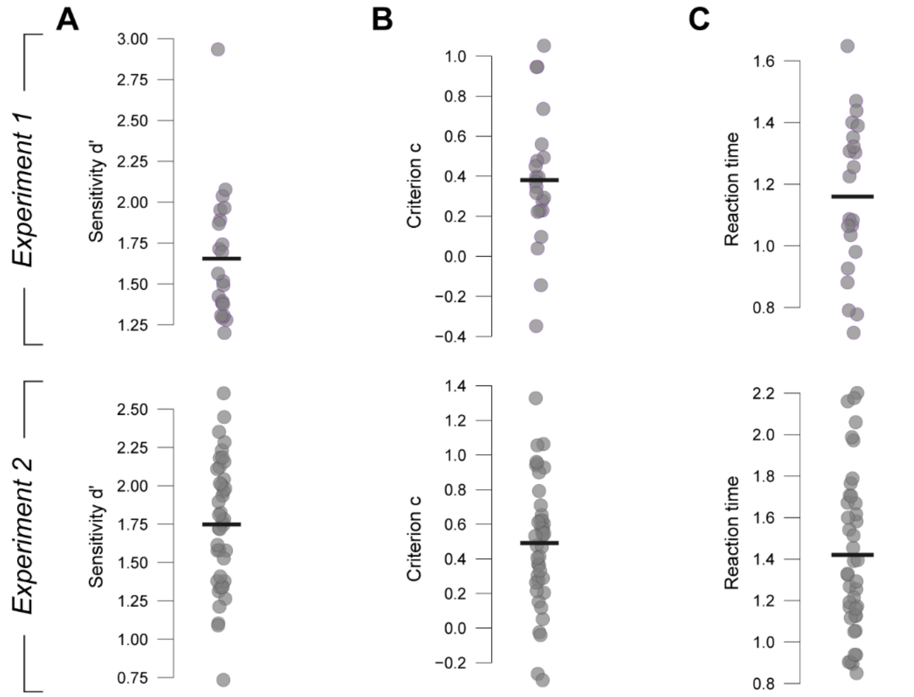

Figure S1. Mean behavior. (A) Mean individual decision performance values (sensitivity d', slope of psychometric function) for Experiments 1 (mean ± S.E.M: 1.655 ± 0.085) and 2 (1.748 ± 0.065). Black bar, group mean. **(B, C)** As A, but for choice bias (mean criterion c ± S.E.M.: Experiment 1, 0.381 ± 0.072; Experiment 2, 0.491 ± 0.056; B) and reaction time (mean ± S.E.M.: Experiment 1, 1.16 ± 0.054 s; Experiment 2, 1.42 ± 0.06 s; C).

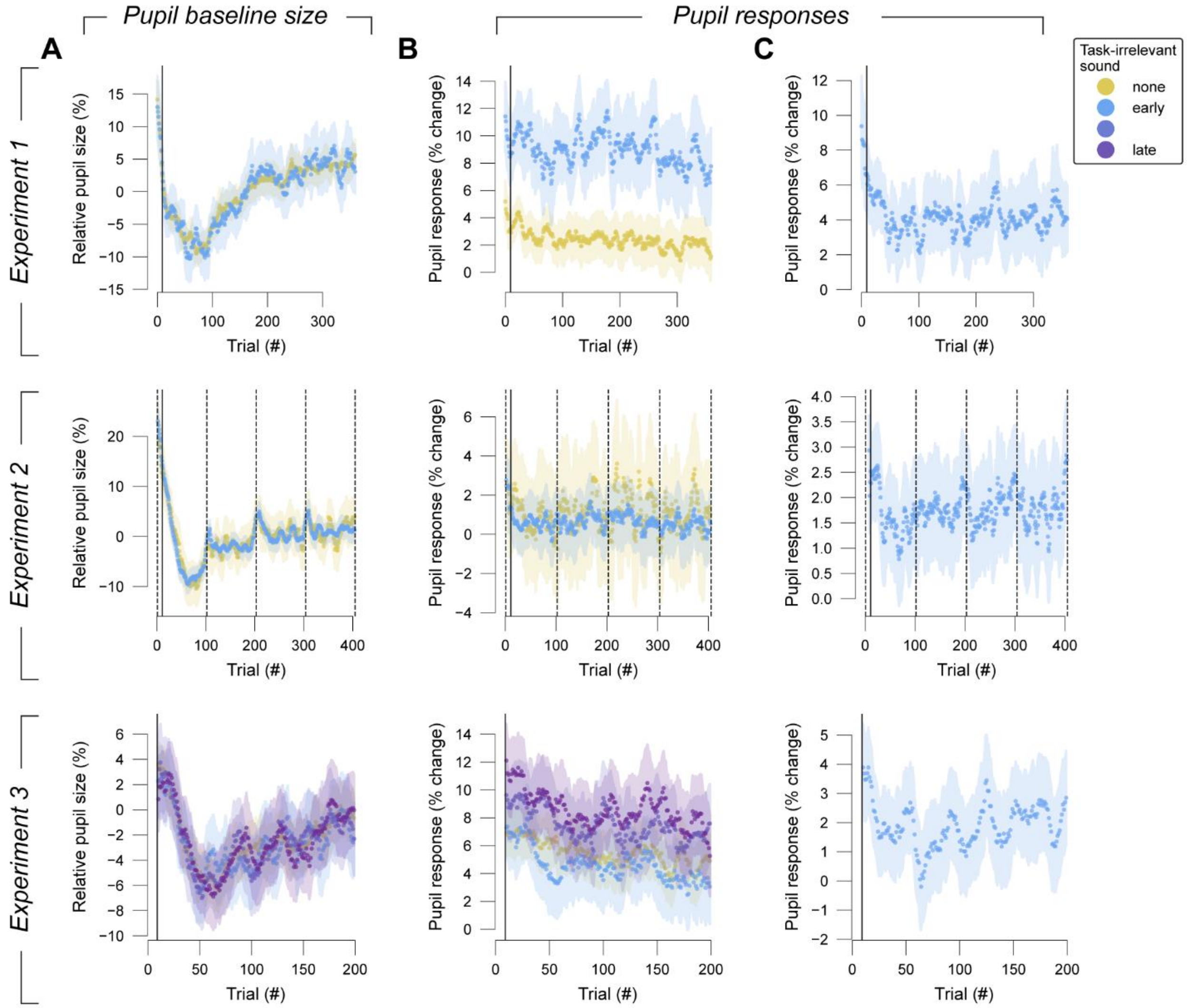

Figure S2. Temporal evolution of baseline pupil sizes, task-irrelevant sound-evoked pupil responses, and task-evoked pupil responses across the experiments. All three measures are shown as a sequence of group-average scalar amplitude values per trial. **(A)** Pre-trial pupil baseline per trials on all blocks by experiment, expressed as percent modulation around the median pupil size across the complete recording. Shaded area, S.E.M. across participants. Line, trial cut-off used for analyses. Dashed line, 10 s-break interval in Experiment 2. **(B, C)** As A, but for task-evoked pupil response (B) and task-irrelevant sound-evoked pupil response (C). Pupil response amplitudes are expressed as percent change relative to pre-trial baseline.

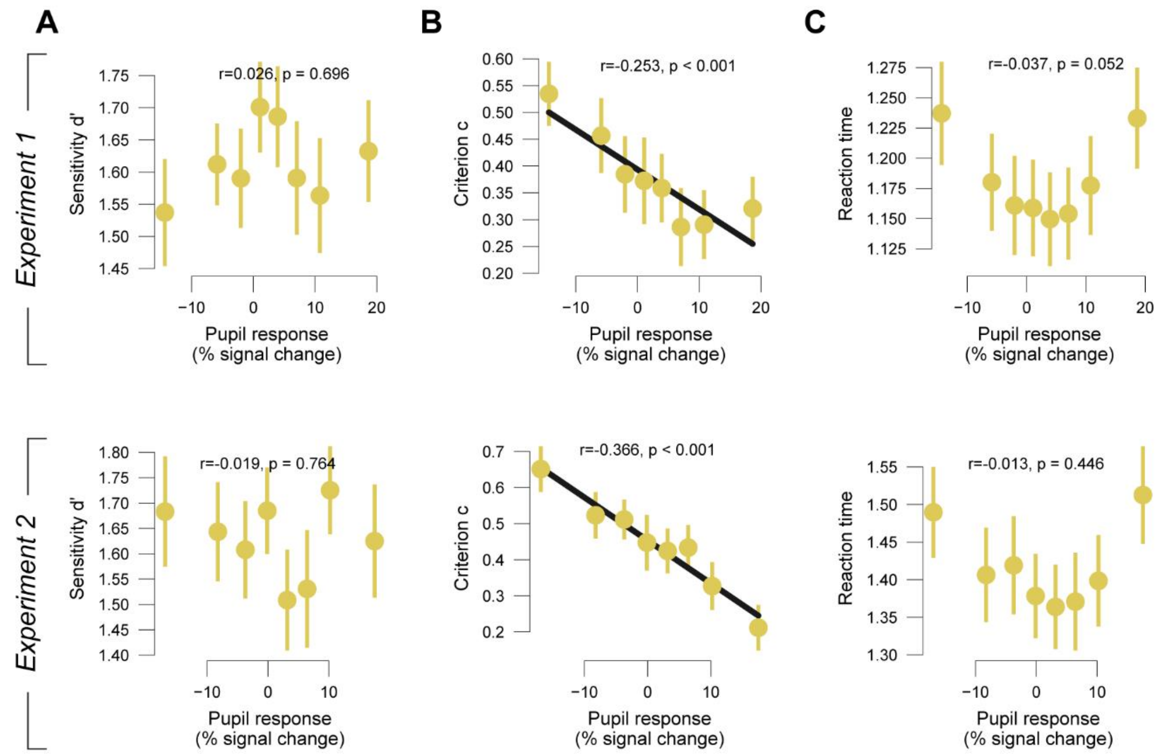

Figure S3. Correlation analyses between TPR and behavior. (A) Decision sensitivity d’ plotted against task-evoked pupil response for experiments 1 and 2. Experiment 3 was not included in this analysis. Error bars, S.E.M. across participants. Only trials with no sound were used for these analyses. **(B, C)** As A, but for choice bias (criterion c; B) and reaction time (C).

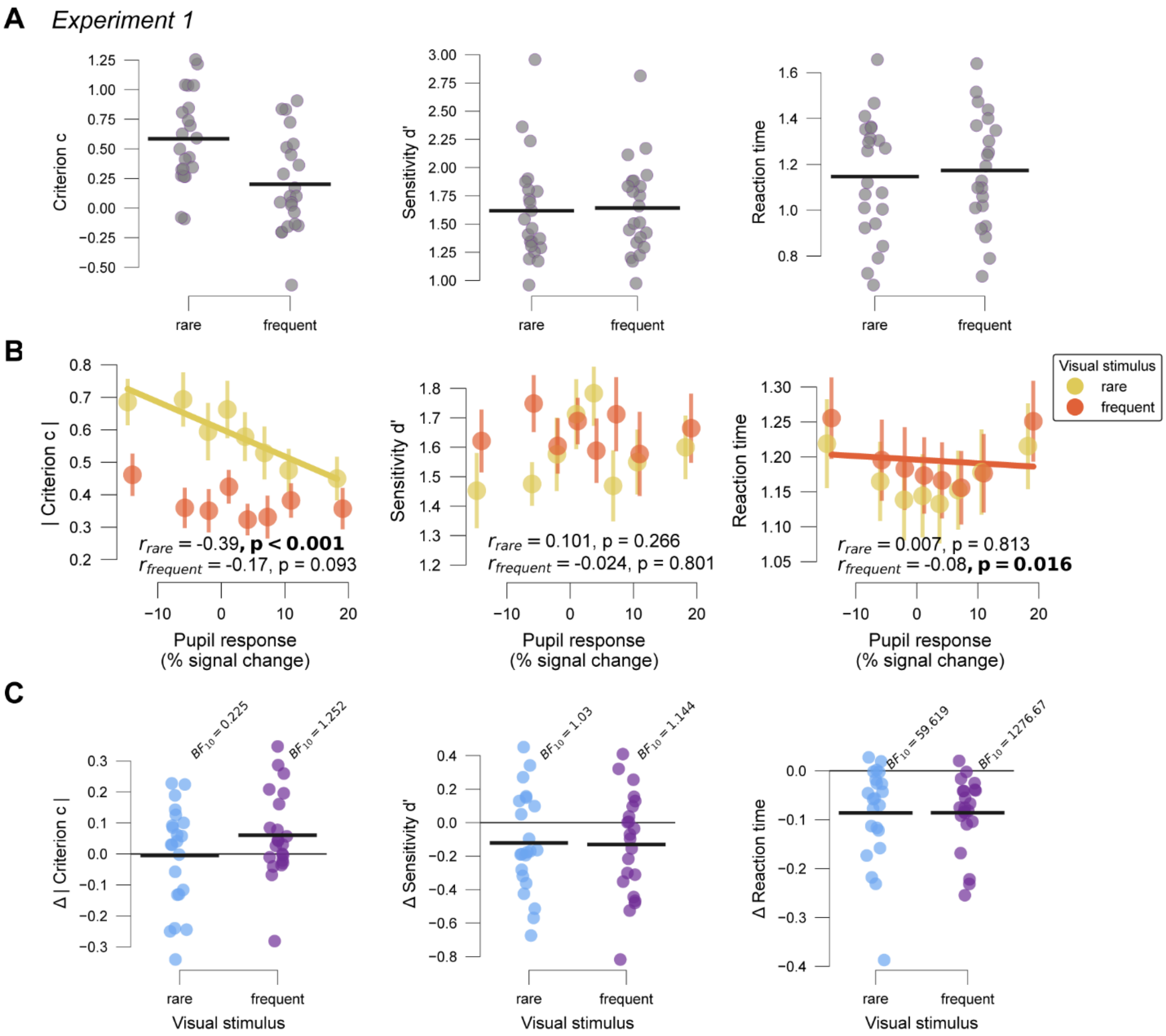

Figure S4. Experiment 1 Behavioral effects during task-evoked pupil responses and of task-irrelevant sound by block type. (A) Mean individual decision bias (criterion c), sensitivity (d') and reaction time values by block type. Black bar, group mean. **(B)** Absolute criterion c, sensitivity d' and reaction time plotted against task-evoked pupil response. Colors represent block type. Error bars, S.E.M. across participants. Only trials without task-irrelevant sound were used for these analyses. **(C)** Individual mean differences in absolute criterion c, sensitivity d' and reaction time between trials with and without task-irrelevant sound. Black bar, mean. BF_{10} values for Bayesian t-tests. Colors represent block type.

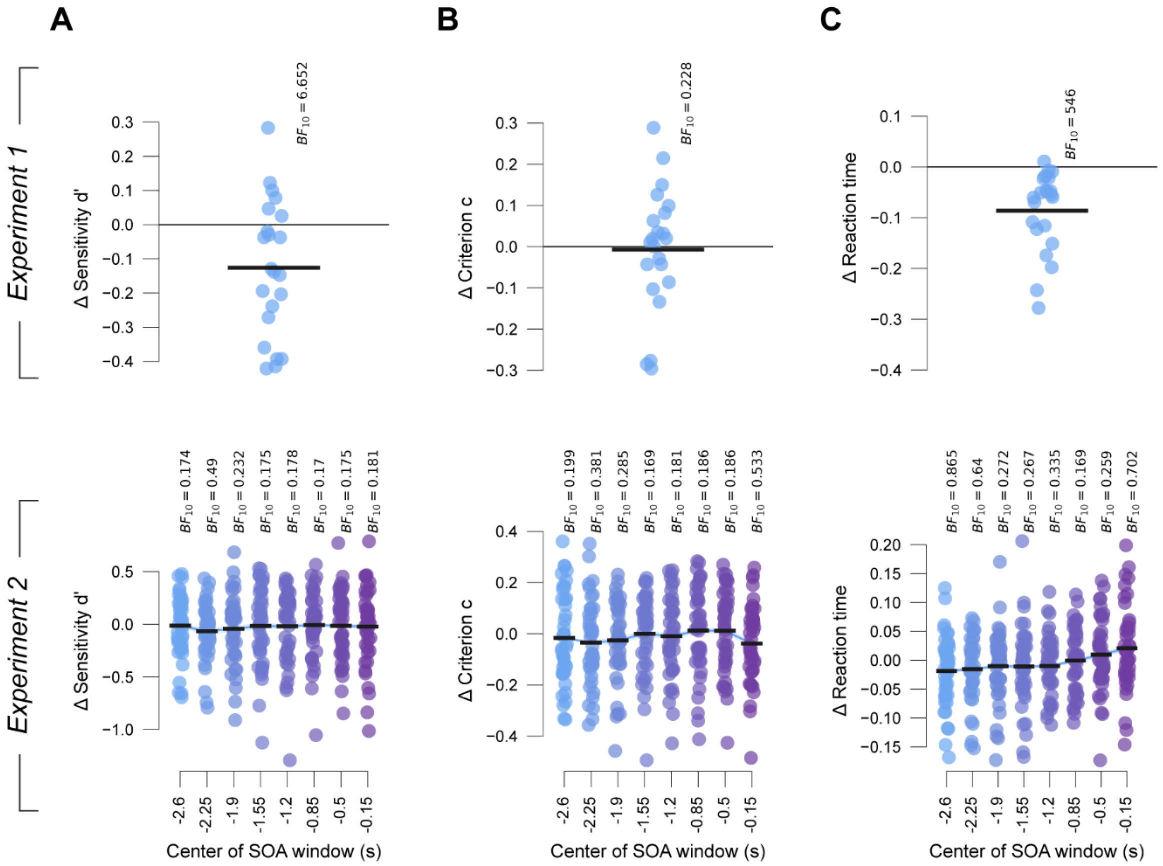

Figure S5. Behavioral responses to task-irrelevant sound. (A) Individual mean differences in sensitivity (d') between trials with and without task-irrelevant sound. Black bar, mean. BF_{10} values for Bayesian t-tests. **(B, C)** As A, but for bias (criterion c) and reaction time, respectively.

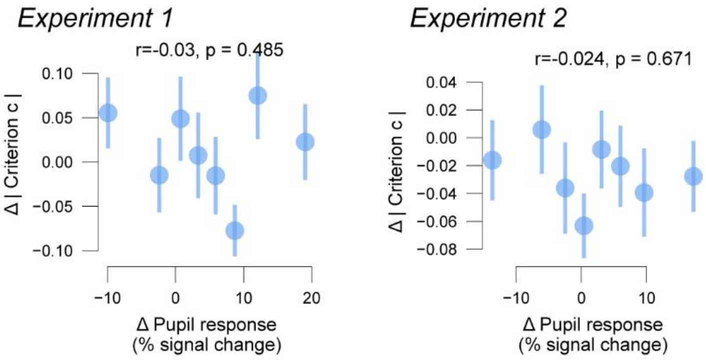

Figure S6. Variability of task-irrelevant sound-evoked pupil response. Absolute bias (criterion c) plotted against pupil response to the task-irrelevant sound. Error bars, S.E.M. across participants.

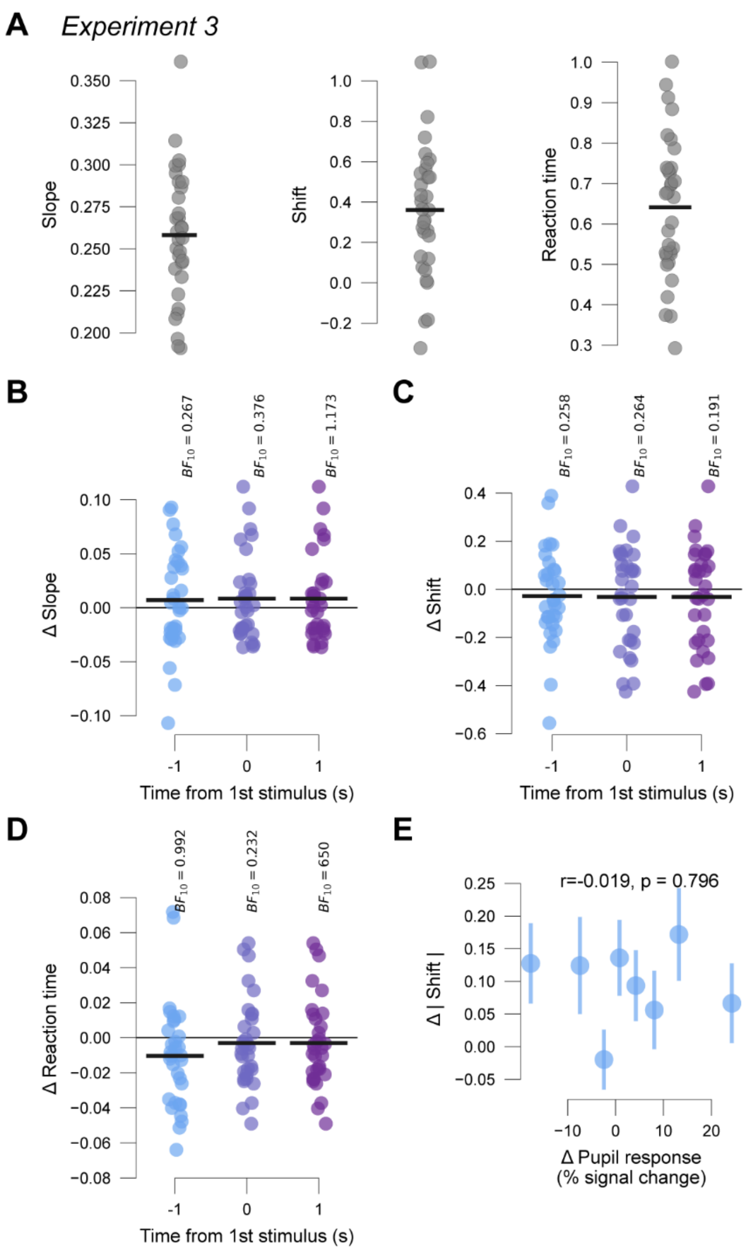

Figure S7. Experiment 3 behavioral means and task-irrelevant sound-evoked effects. (A) Mean participant-wise performance (mean slope of psychometric function ± S.E.M. across participants: 0.258 ± 0.007), choice bias (mean shift of psychometric function ± S.E.M. across participants: 0.36 ± 0.059) and reaction time (mean ± S.E.M. across participants: 0.642 ± 0.031 s). Black bars, overall mean across participants. **(B)** Individual mean differences in performance between trials with and without task-irrelevant sound. Black bar, mean. BF_{10} values for Bayesian t-tests. Colors represent different task-irrelevant sound timing conditions. **(C, D)** As B, but for choice bias and reaction time, respectively. **(E)** Absolute bias plotted against pupil response to the task-irrelevant sound. Error bars, S.E.M. across participants.
